# YARP-ROS Inter-Operation in a 2D Navigation Task

**DOI:** 10.3389/frobt.2018.00005

**Published:** 2018-02-16

**Authors:** Marco Randazzo, Andrea Ruzzenenti, Lorenzo Natale

**Affiliations:** ^1^iCub Facility, Istituto Italiano di Tecnologia, Genova, Italy

**Keywords:** YARP, autonomous navigation, SLAM, mobile robots, iCub, R1, ROS, C++ interfaces

## Abstract

This paper presents some recent developments in YARP middleware, aimed to improve its integration with ROS. They include a new mechanism to read/write ROS transform frames and a new set of standard interfaces to intercommunicate with the ROS navigation stack. A novel set of YARP companion modules, which provide basic navigation functionalities for robots unable to run ROS, is also presented. These modules are optional, independent from each other, and they provide compatible functionalities to well-known packages available inside ROS framework. This paper also discusses how developers can customize their own hybrid YARP-ROS environment in the way it best suits their needs (e.g., the system can be configured to have a YARP application sending navigation commands to a ROS path planner, or vice versa). A number of available possibilities is presented through a set of chosen test cases applied to both real and simulated robots. Finally, example applications discussed in this paper are also made available to the community by providing snippets of code and links to source files hosted on github repository https://github.com/robotology.^1^

## 1 Introduction

YARP is an open-source robotics middleware, specifically designed to be modular, non-invasive, and flexible. It promotes software re-usability by means of abstract interfaces and modular software paradigms, and it allows to distribute computational tasks across a system by offering multi-platform network communication primitives (Fitzpatrick et al., 2014).

YARP development is historically correlated to the iCub robot (Metta et al., 2010; Natale et al., 2016), a child-sized humanoid platform for the study of cognitive robotics. In these years, the iCub community focused its attention on topics such as human–robot interaction, visual attention, machine learning, object manipulation, and grasping. Balancing a bipedal walking robot like iCub is a problem that has been addressed only recently by some research groups (Hu et al., 2016; Nava et al., 2016). This is the reason why a standard navigation interface was missing in YARP so far.

On the other side, ROS, an Ubuntu-based middleware developed around the PR2 wheeled robot, addressed the problem of making a mobile platform to navigate into a 2D environment from the very beginning (Quigley et al., 2009; Cousins, 2010). Over the past years, the ROS navigation stack has grown in comprehensiveness, wrapping or including bindings to basically all state-of-the-art algorithms and third-party libraries (Marder-Eppstein et al., 2010).

This paper has two goals. First, to provide the YARP community a way to re-use the massive amount of code that has been developed within the ROS community. Second, Yarp is a multi-platform framework which can run on Windows, Linux and MacOs, while ROS is currently limited to Ubuntu-based systems. Thus, Yarp can be used to interface applications belonging to the two different frameworks and running on different operating systems. This goal is accomplished through a set of dedicated YARP classes and interfaces, as shown in the following sections.

## 2 YARP/ROS Interface

### 2.1 YARP Ports and ROS Topics

YARP inter-module communication is traditionally implemented through network objects called *ports*. In a typical usage scenario, a sender module opens an output port (identified by a symbolic name, registered onto a nameserver) and writes data to it. Analogously, a receiver module, which wants to perform a read operation, opens an input port with a different symbolic name. Sender and receiver are thus decoupled, and the user is responsible for making connections/disconnections between the two ports.

In ROS, inter-module communication is obtained through a *publisher/subscriber* paradigm, based on the concept of *topic*. The subscriber manifests its intention of receiving a specific stream of data by registering to a topic, without caring about the identity of the *node* (or nodes) that is actually publishing it. Connections are not managed by the user but by a central authority, called *ROS Master*, which also checks if publishers and receivers comply on the same data format. Indeed, ROS communication is strongly typed and it employs a set of standard formats defined in message (.msg) files.

The possibility to communicate natively with ROS has been recently integrated into YARP. Special classes such as yarp::os::Node, yarp::os::Publisher, and yarp::os::Subscriber have been introduced to allow a user to handle ROS topics. Additionally, a specialized converter, namely *yarpidl_rosmsg*, was developed to automatically generate C++ header files from ROS.msg files and to allow the usage of ROS data types inside YARP.

An example of a YARP module directly publishing data onto a ROS topic, without linking any external ROS library, is shown in Section I in Supplementary Material.

### 2.2 TransformServer and TransformClient

Tf is a ROS package which allows a distributed system to keep track of multiple coordinate frames over time. For example, a module may be able to compute and publish the transformation from reference frame /*a* to reference frame /*b* while a different module may be able to publish the transformation from frame /*b* to frame /*c*. By subscribing to the /*tf topic*, a third module can retrieve the broadcasted transforms and compute the resulting transformation from /*a* to /*c*.

This mechanism is pervasive in all ROS. Remarkable application examples are *move-it* (a motion planning framework for mobile manipulation), *Rviz* (a 3D visualization tool), and the *ROS navigation stack*. In this latter case, tf is typically used to keep track of the estimated robot position with respect to an odometry reference frame or to a map origin. Thus, it is clear that it is not possible to obtain a complete YARP-ROS integration without implementing a mechanism that is able to handle ROS frame transforms in YARP.

**Figure 1:**
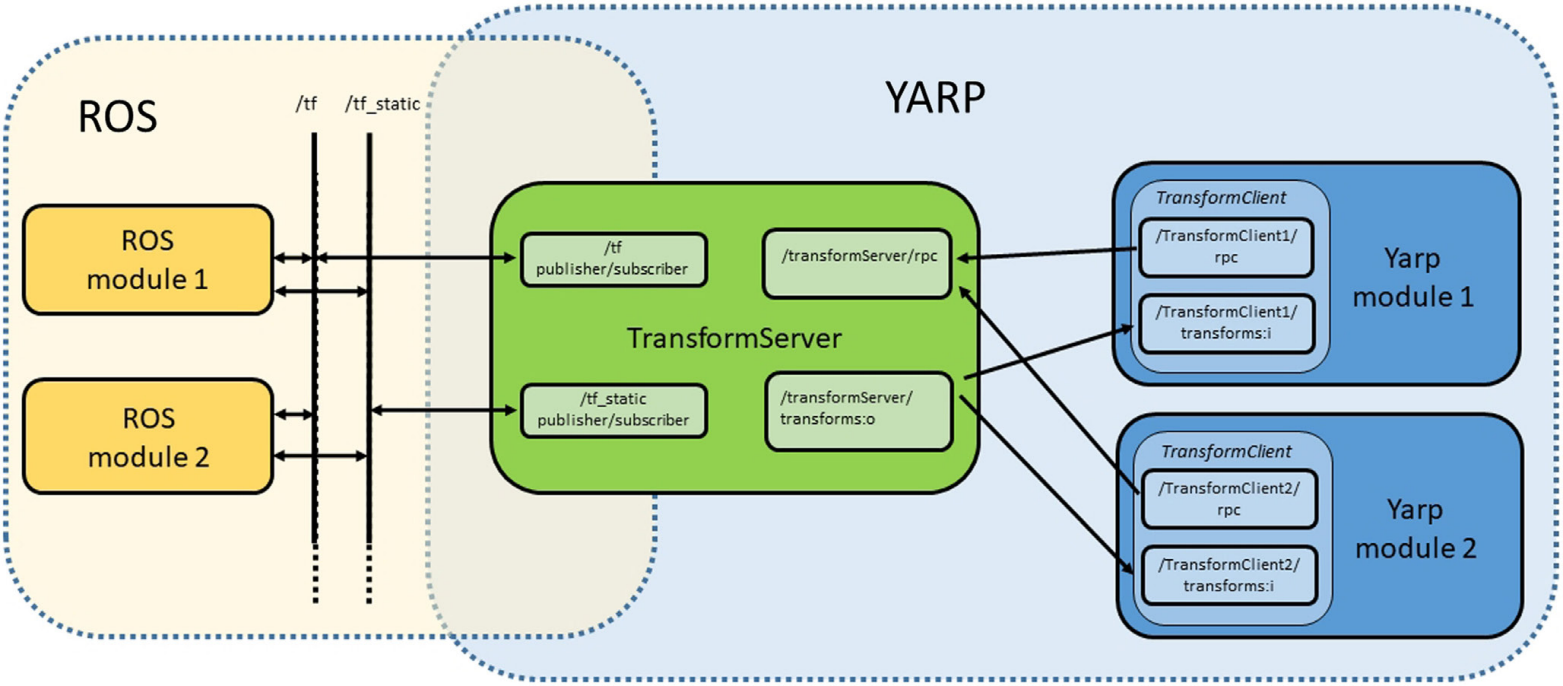
Typical scenario in which multiple YARP modules, each of them instantiating its own yarp::dev::transfomClient, communicate with a single yarp::dev::transformServer. The latter is responsible for synchronizing YARP transforms with ROS data, publishing and subscribing to /*tf* and /*tf_static* topics.

To overcome this limitation, we developed a YARP device called *transformServer*. *TransformServer* collects and stores frame transforms by subscribing to /*tf* and /*tf_static* topics and makes these information available to a YARP *transformClient* instance inside a user module (Figure 1). TransformClient is an entity which implements the yarp::dev::IFrameTransform interface (see Sections II and III in Supplementary Material). Available methods allow the user to query the server about the registered YARP and ROS transforms, to perform kinematic computations, and to register on the server new transforms computed by YARP modules.

## 3 YARP Classes and Interfaces for Navigation

This section presents the new YARP classes and interfaces specifically designed for managing maps and controlling a robot during a navigation task. Detailed description of available methods and usage examples are shown in Supplementary Material.

### 3.1 MapGrid2D

The class yarp::dev::MapGrid2D is the main YARP class used to store map data. Similar to ROS occupancy grid message (*nav_msgs/OccupancyGrid.msg*), data are organized in square cells of fixed size (e.g., 0.05 m × 0.05 m), each of them storing the probability of being occupied by a fixed obstacle (e.g., a wall). This information is typically used to localize the robot in an environment previously mapped by a SLAM algorithm. In addition to this property, map cells are also provided with an additional flag (Section IV in Supplementary Material), which can be used to control the robot behavior. For example, a user can choose to set keep-out areas, which should be avoided by the robot when it computes its path, or critical areas in which the robot should stop when an obstacle is encountered (instead of finding an alternate path). Finally, MapGrid2D is equipped with methods to save/load maps both in YARP and in a ROS compatible format.

### 3.2 Map2DLocation

A yarp::dev::Map2DLocation is a support class used to store user location information. A location is composed of the location name, the map name to which the location refers to, and the (x,y,*θ*) coordinates w.r.t. the map origin. Locations are typically stored together with maps in a *map2DServer* (see Section 4.1) so that a user can invoke the navigation APIs using the location name instead of the corresponding coordinates. Locations are also used by *map2DServer* to define interconnection points between multiple YARP maps.

### 3.3 IMap2D

yarp::dev::IMap2D is a pure virtual interface dedicated to the management of MapGrid2D and Map2DLocation entities. A *Map2DServer* (Section 4.1) implements methods of this interface to satisfy the requests from a *Map2DClient*. The complete listing of the methods belonging to yarp::dev::IMap2D as well as an application example is shown in Sections V and VI in Supplementary Material.

### 3.4 INavigation2D

yarp::dev::INavigation2D is a pure virtual interface shared between all client/server modules, which performs navigation tasks. The most classical usage in a user application requires the instantiation of a yarp::dev::INavigation2DClient to send navigation commands to the robot (e.g., “go to the entrance room”). On the other side, the server counterpart, which can be any module implementing the same yarp::dev::INavigation2D interface (e.g., robotPathPlanner, see Section 4.6), receives the goal command and computes the path required by the robot to reach the goal.

*INavigation2D* contains methods to start, pause, and resume navigation tasks, both in absolute (with respect to the map reference frame) or in relative coordinates (with respect to the robot reference frame) (Section VII Supplementary Material). Additionally, it allows the user to assign names to the current robot position and to important locations on the map. These names might be used instead of absolute coordinates when commanding a goal to the robot. Finally, the user can query the current status of the navigation task. The enum returned by the method INavigation2D::getNavigationStatus() can be used by the client application to know when the goal has been reached or if a problem occurred (Section VIII in Supplementary Material).

## 4 YARP Modules and Tools for Navigation

This section describes the YARP modules and tools which constitute the core of the YARP navigation suite. They are provided inside robotology/yarp and robotology/navigation github repositories. A comparison between these YARP tools and similar ones provided by ROS is reported in Table 1.

**Table 1:** Similarities and correspondences between YARP and ROS modules with similar functionalities.

YARP	ROS	Notes
Map2DServer	map_server	map_server offers a single map via ROS latched topic/map. Map2DServer acts a storage for multiple maps and user-defined locations

BaseControl	–	In ROS, there is no equivalent module. Each kind of robot exposes its own specific control interface

Mapper2D	gmapping	gmapping performs loop closure detection and simultaneous localization and mapping. Mapper2D allows to set not only the occupancy value of the cell but also the YARP map flag

LocalizationServer	–	LocalizationServer does not have a direct correspondence in ROS. It acts as a bridge for a ROS localization module like Adaptive Montecarlo Localization (AMCL) adding the support for YARP map collections (not directly supported in ROS)

–	AMCL	YARP navigation suite currently does not provide any localization system for mobile robots. A YARP user may use a ROS module such as AMCL to estimate the robot position against a known map or use its own localization system

RobotGoto	move_base-base_local_planner	The two modules have similar functionalities although ROS base_local_planner supports multiple algorithms (e.g., Trajectory Rollout and Dynamic Window Approach) while RobotGoto artificial potential fields approach is more tailored to work together with YARP RobotPathPlanner

RobotPathPlanner	move_base-global_planner	The two modules have similar functionalities and use comparable algorithms

### 4.1 Map2DServer

*Map2DServer* implements the methods of the YARP interface yarp::dev::IMap2D, and it allows a client application (such as the navigation module) to store and retrieve maps (yarp::dev::MapGrid2D) from memory. It can be initialized at startup by a map collection file which contains an index of all map files to be used in the session. It must be noticed that this module only behaves as a storage, and it contains neither information about the current robot position nor the name of the map in which the robot finds itself. These tasks are performed by other modules (e.g., *localizationServer*, Section 4.4) which interact with the map2Dserver when they need to obtain map data. Finally, this module implements some methods of the yarp::dev::INavigation2D interface, allowing to store/retrieve user notable locations (yarp::dev::Map2DLocation) on a map.

### 4.2 BaseControl

*BaseControl* is the core YARP module used to control a mobile robot. It receives cartesian velocity commands ($\dot{x},\;\dot{y},\;\dot{\theta }$) either from a navigation module or from a joystick device, and it computes the corresponding actuators actions required to achieve them. *BaseControl* is also responsible for computing robot odometry, i.e., estimating the robot position in the world using measured motions of robot actuators. Computed data are published on a YARP port both as a vector (x,y,*θ*) and, via *transformClient*, as a transform between the origin of the odometry system (*/odom*) and the robot (*/mobile_base*). This allows a ROS module to interface with the robot by subscribing to the /*tf* topic.

### 4.3 Mapper2D

*Mapper2D* is a simple YARP module which registers laser scans to build an occupancy-based map. The module is not equipped with a loop closure detector, nor with an internal localization algorithm; thus, it is not suitable to perform stand-alone SLAM tasks. Instead, it is designed to receive accurate localization data from an external source (e.g., a Google Tango device) either via YARP port or via *transformClient*.

### 4.4 LocalizationServer

*LocalizationServer* is an auxiliary tool which acts as the server side of a *Navigation2DClient* for the INavigation2D::getCurrentPosition() and INavigation2D::setInitialPose()methods. *Robotology/navigation* repository does not provide a default localization system for a mobile robot. A YARP user may thus choose to employ a YARP-based localization system (such as *Robust-View-Graph-SLAM*), or a ROS-based one (e.g., *AMCL, RTAB-Map, Tango*-*ROS*-*Streamer*). In this latter case, *LocalizationServer* acts as a bridge between the ROS world (which is single map) and the YARP world (which is multi-map). When the user sets an initial position to initialize the localization algorithm, it specifies a yarp::dev::Map2DLocation which is translated to a string (the map name, handled by the *Map2DServer*) and a (x,y,*θ*) vector. This latter is sent with a *geometry_msgs/PoseWithCovarianceStamped* message to the ROS localization module as the estimated robot pose with respect to the origin frame of the current map.

### 4.5 RobotGoto

This module computes the cartesian velocities ($\dot{x},\;\dot{y},\;\dot{\theta }$) of the mobile base required to reach the commanded goal, given the current robot position (provided through a *transformClient*) and a set of parameters that controls the trajectory generation (e.g., differential drive or holonomic robot kinematics, heading and goal tolerance, etc.).

*RobotGoto* does not use any map information, except for the local occupancy grid which is continuously updated according to sensor data. An artificial potential field algorithm is employed to allow the robot to avoid obstacles obstructing the path to the goal. Depending on the configuration parameters, if a deadlock is detected, navigation may be paused (waiting a human to remove the obstacle) or aborted. In this latter case, the high-level path planner is notified by a specific yarp::dev::INavigation2D::NavigationStatusEnum, as shown in Section VIII and Figure S1 in Supplementary Material.

### 4.6 RobotPathPlanner

This module is responsible for generating the navigation waypoints to be pursued by a local navigation module (e.g., *robotGoto*). By implementing the INavigation2D interface, *robotPathPlanner* acts as the server counterpart of a *Navigation2DClient* instantiated by a user module. For example, when the user calls the INavigation2D::gotoAbsolutePosition() method to command the robot to reach a new goal, *robotPathPlanner* becomes in charge of performing the navigation task, notifying the user about its current status (e.g., in progress, goal reached, etc.).

The algorithm acts as follows. *RobotPathPlanner* retrieves from a *Map2DServer* instance the current map of the area. A valid path from the current robot location to the goal is computed using A* algorithm. If the path does not exists, navigation is aborted. Otherwise the path, initially defined as a vector of map cells, is transformed into a sequence of navigation waypoints. To be accepted, these waypoints must satisfy some user-defined parameters (e.g., minimum distance between the cells etc.). Waypoints are then put in a queue and sent one by one to a local navigation algorithms (such as *robotGoto*) which will pursue them.

*RobotPathPlanner* is also responsible for processing the YARP flags assigned to particular areas of the map. These flags may belong to two different categories. Those which alter the navigation trajectory (such as keep-out areas) are directly processed by the module during the trajectory generation phase. Instead, flags which alter the robot behavior (e.g., areas in which the robot must proceed at a different speed or interrupt the navigation if an obstacle is detected on the path) are not directly processed. Indeed, since they affect the behavior of the local navigation task, a proper set of commands is generated and sent to *RobotGoto* to modify the default navigation parameters.

Finally, *RobotPathPlanner* is able to show the computed robot trajectory by means of the standard YARP graphical visualization tool *yarpview* and, additionally, to receive navigation commands from it (dragging an arrow on the map will be interpreted as goal command).

## 5 Navigation Integration and Examples

YARP and ROS may inter-operate in several ways to attain a navigation task. Different possibilities range from using a full YARP-based framework to using the complete ROS navigation stack. In between there exist a number of possible combinations: as shown in previous sections, most of the YARP components can be replaced by a ROS equivalent or vice versa, depending on the user needs and preferences.

**Figure 2:**
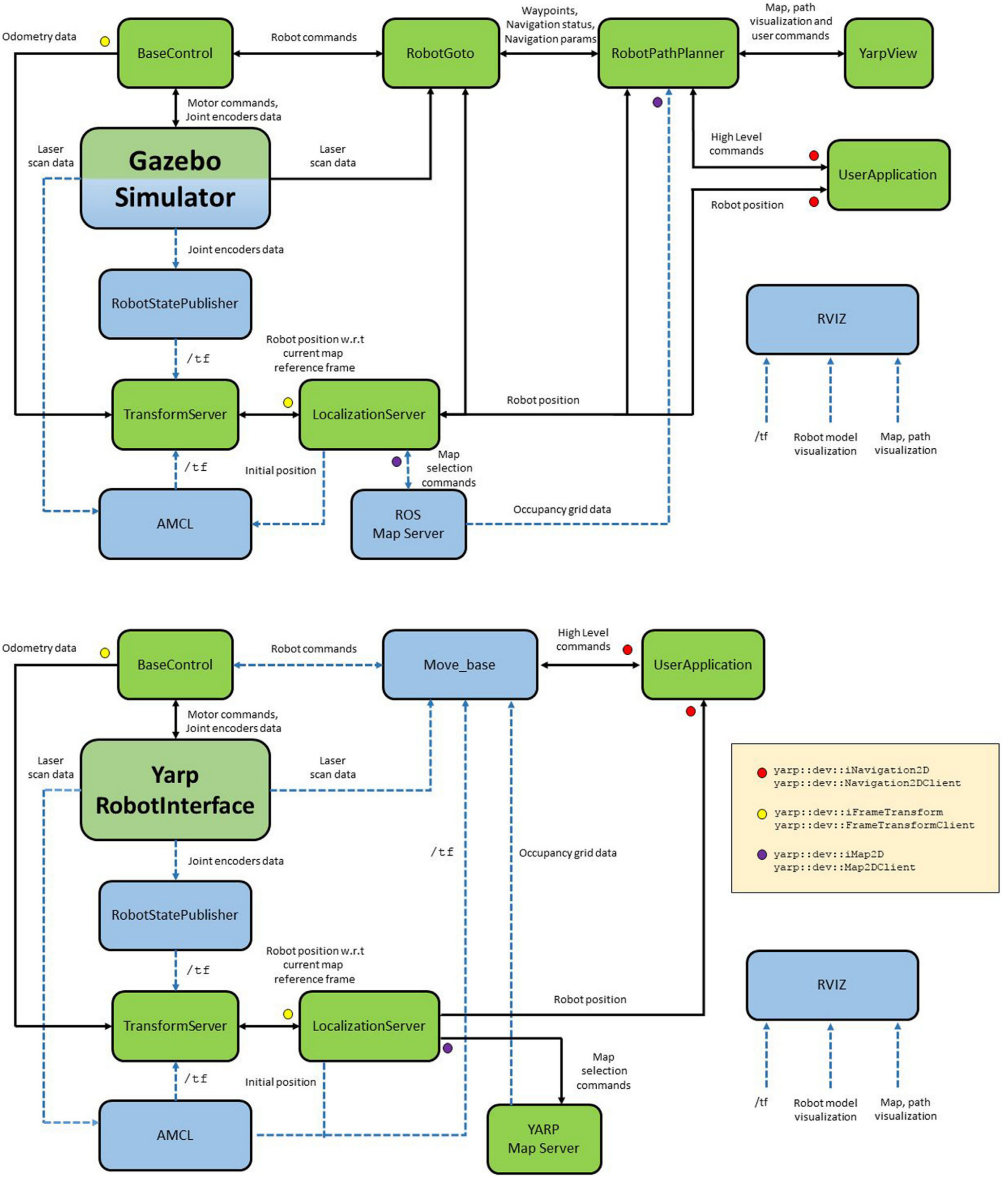
Two realistic application scenarios, in which different combinations of YARP (green) and ROS (blue) modules are employed. Solid lines represent YARP port connections. Dashed lines represent ROS topic connections (Rviz connections are omitted for diagram clearness). Colored markers indicate the YARP interfaces employed to interconnect the various client/server modules. Gazebo simulator is represented as a hybrid YARP/ROS module because its modular design allows to execute plugins belonging to both frameworks (Mingo Hoffman et al., 2014).

Figure 2 shows two illustrative scenarios. The first example refers to a simulated wheeled robot in *Gazebo*, a generic, multi-robot, physics simulator. The navigation task is carried out by robotGoto/robotPathPlanner modules. Since ROS *map_server* is used, robotPathPlanner employs only the occupancy grid information and no YARP map flags are available.

The second example refers to a real wheeled robot (i.e. R1 (Parmiggiani et al., 2017)) controlled by *yarpRobotInterface*, the core YARP application which manages the low-level hardware control. In this case, navigation task is carried out by ROS navigation stack encapsulated inside *move_base* node.

It must be noticed that, in both scenarios, the final end-user is a YARP application which instantiate a yarp::dev::INavigation2DClient. Section IX in Supplementary Material shows a simple application which controls the robot to reach a location stored into the map server, unaware of which framework and control modules are employed underneath. The included sequence diagram (Figure S2 in Section X in Supplementary Material) shows the timing and the messages exchanged between the clients opened by the example and the connected external modules (i.e., LocalizationServer, Map2DServer, robotPathPlanner).

Finally, a set of examples of increasing complexity is included in the github repository (Figure S3 in Section X in Supplementary Material), as well as some skeleton applications which the user can exploit to develop its own navigation modules.

## 6 Conclusion and Future Work

In this paper, we showed latest developments to improve YARP interoperability with ROS. These improvements allow a robotics developer to use YARP middleware without giving up popular and convenient ROS features, such as the /tf package. By introducing a brand new set of standard interfaces, such as yarp::dev::IMap2D and yarp::dev::INavigation2D, YARP is now capable of performing a 2D navigation task, natively or interacting with ROS.

Future work will be aimed to further improve YARP-ROS integration. YARP transformServer is currently unable to interpolate/extrapolate frames over time, an advanced feature that is instead available in the ROS /tf package, which allows users to ask for the pose of a frame at a specific time instant, in the past or even in the future. Additionally, YARP is currently unable to manage octomaps or other 3D data types. Their introduction is thus a required step to allow foot planning of a bipedal robot on a highly structured terrain.

## Author Contributions

MR: development of YARP interfaces and classes for navigation; development of the navigation modules belonging to https://github.com/robotology/navigation repository; and experiments with real and simulated robots. AR: development of transformServer/transformClient, development of automatic tests for frameTransform and navigation interfaces; and experiments with real and simulated robots. LN: development of YARP framework and scientific supervision.

## Conflict of Interest Statement

The authors declare that the research was conducted in the absence of any commercial or financial relationships that could be construed as a potential conflict of interest.
