# Supplementary material for: YARP-ROS Inter-Operation in a 2D Navigation Task
Source: Front Robot AI. 2018 Feb 16;5:5. doi: 10.3389/frobt.2018.00005 (PMC7805980; doi:10.3389/frobt.2018.00005)
Supplement: Supplementary file 1 [file Supplementary_Material.pdf]

Supplementary Material: YARP-ROS inter-operation in a 2D navigation task

Marco Randazzo *, **Andrea Ruzzenenti, and Lorenzo Natale**

*Correspondence:
Marco Randazzo
marco.randazzo@iit.it

SUPPLEMENTARY MATERIAL

I PUBLISHER/RECEIVER EXAMPLE

The following code snippet shows an example of a YARP module publishing data onto a ROS topic.

```
#include <iostream>
#include <yarp/os/all.h>
using namespace yarp::os;
using namespace std;

// Make sure you run yarpidl_rosmsg std_msg/String to generate String.h
#include "String.h"

int main(int argc, char *argv[])
{
    Network yarp;

    /* creates a node called /yarp/talker */
    Node node("/yarp/talker");

    /* subscribe to topic chatter */
    yarp::os::Publisher<String> publisher;
    if (!publisher.topic("/chatter"))
    {
        cerr<< "Failed␣to␣create␣publisher␣to␣/chatter\n";
        return -1;
    }
    while (true)
    {
        /* prepare some data */
        String data;
        data.data="Hello␣from␣YARP";
        /* publish it to the topic */
        publisher.write(data);
        /* wait some time to avoid flooding with messages */
        Time::delay(0.1);
    }
    return 0;
}
```

The following code snippet shows an example of a YARP module subscribing to a ROS topic to receive data.

```
#include <iostream>
#include <yarp/os/all.h>
using namespace yarp::os;
using namespace std;

// Make sure you run yarpidl_rosmsg std_msg/String to generate String.h
#include "String.h"

int main(int argc, char *argv[])
{
    Network yarp;

    /* creates a node called /yarp/listener */
    Node node("/yarp/listener");

    /* subscribe to topic chatter */
    yarp::os::Subscriber<String> subscriber;
    if (!subscriber.topic("/chatter"))
    {
        cerr<< "Failed␣to␣subscriber␣to␣/chatter\n";
        return -1;
    }
    /* read data from the topic */
    while (true)
    {
        String data;
        subscriber.read(data);
        cout << "Received:" << data.data << "␣" << endl;
    }
    return 0;
}
```

II IFRAMETRANSFORM INTERFACE

A quick reference to the methods belonging to the `yarp::dev::IFrameTransfom` interface is shown below (see http://www.yarp.it/classyarp_1_1dev_1_1IFrameTransform.html for a complete documentation).

`yarp::dev::FrameTransformClient` inherits from `yarp::dev::IFrameTransfom`. It implements the methods required to communicate with a `yarp::dev::FrameTransformServer`.

```
class yarp::dev::IFrameTransform
{
    public:
    enum
    {
        TRANSFORM_OK            = 0,
        TRANSFORM_GENERAL_ERROR = 1,
        TRANSFORM_TIMEOUT       = 2,
    };
    bool allFramesAsString(string &all_frames) = 0;
    bool canTransform (const string &target_frame, const string &source_frame)  = 0;
```

```
    bool clear () = 0;
    bool frameExists (const string &frame_id) = 0;
    bool getAllFrameIds (vector< string > &ids) = 0;
    bool getParent (const string &frame_id, string &parent_frame_id) = 0;
    bool getTransform (const string &target_frame_id, const string &source_frame_id, Matrix &transform)
        = 0;
    bool setTransform (const string &target_frame_id, const string &source_frame_id, const Matrix &
        transform) = 0;
    bool setTransformStatic(const string &target_frame_id, const string &source_frame_id, const Matrix &
        transform) = 0;
    bool deleteTransform (const string &target_frame_id, const string &source_frame_id) = 0;
    bool transformPoint (const string &target_frame_id, const string &source_frame_id, const Vector &
        input_point, Vector &transformed_point) = 0;
    bool transformPose(const string &target_frame_id, const string &source_frame_id, const Vector &
        input_pose, Vector &transformed_pose) = 0;
    bool transformQuaternion(const string &target_frame_id, const string &source_frame_id, const yarp::
        math::Quaternion &input_quaternion, yarp::math::Quaternion &transformed_quaternion) = 0;
    bool waitForTransform(const string &target_frame_id, const string &source_frame_id, const double &
        timeout) = 0;
};
```

III TRANSFORMCLIENT EXAMPLE

The following code snippet, extracted from YARP FrameTransformClient regression test, shows an example in which a `yarp::dev::FrameTransformClient` registers on the `yarp::dev::FrameTransformServer` two transforms: *frame1*→*frame2* and *frame2*→*frame3*. The client is then asked to compute the chained transform *frame1*→*frame3*. The server needs to be externally launched before the execution of this example.

```
#include <yarp/os/all.h>
#include <yarp/dev/IFrameTransform.h>
using namespace yarp::os;
using namespace std;

int main(int argc, char *argv[])
{
    Network yarp;

    //open a transformClient device
    PolyDriver ddtransformclient;
    Property pTransformclient_cfg;
    pTransformclient_cfg.put("device", "transformClient");
    pTransformclient_cfg.put("local", "/transformClientTest");
    pTransformclient_cfg.put("remote", "/transformServer");
    bool ok_client = ddtransformclient.open(pTransformclient_cfg);
    if(!ok_client){return;}

    //get a IFrameTransform interface from the transformClient device
    IFrameTransform* itf = 0;
    bool ok_view = ddtransformclient.view(itf);
    if(!ok_view){return;}
    yarp::os::Time::delay(1.0);
```

```

    yarp::sig::Matrix m1(4, 4); m1.eye();
    yarp::sig::Matrix m2(4, 4); m2.eye();
    yarp::sig::Matrix m3(4, 4); m3.zero();

    //register the two static transforms on the FrameTransformServer
    itf->setTransformStatic("frame2", "frame1", m1);
    itf->setTransformStatic("frame3", "frame2", m2);
    yarp::os::Time::delay(1.0);

    //compute the chained transform m3=m1*m2
    itf->getTransform("frame3", "frame1", m3);
    cout << "Resulting␣transform␣from␣<frame1>␣to␣<frame3>␣is:" << endl;
    cout << m3.toString();
}
```

IV MAP FLAGS ENUM

A list of the flags currently implemented by `yarp::dev::MapGrid2D::map_flags` is shown in the following table. Additional flags can be added by the user.

map_flags	Notes
map_cell_free	The cell is free and the robot can pass though it.
map_cell_keep_out	The cell does not contain any detectable obstacle, but is marked as keep-out, so computed path will avoid this area.
map_cell_temporary_obstacle	The cell is marked as free on the map, but robot sensors detected that it is currently occupied by an obstacle. Temporary obstacles are not used by localization modules to determine robot position.
map_cell_enlarged_obstacle	The cell is marked as free on the map, but path planning algorithm is currently considering it as a keep-out area because a nearby obstacle is potentially able intersect robot footprint. Temporary obstacles are not used by localization modules to determine robot position.
map_cell_wall	The cell is occupied by a fixed wall and it can be used for robot localization.
map_cell_unknown	The cell occupancy status is unknown. Mapping is required.
map_cell_unsafe_area	The robot will stop when an obstacle is detected in this area, instead of trying to avoid it.
map_cell_caution_area	The robot will move with user-definable reduced speed when crossing this particular area.

V IMAP2D INTERFACE

A quick reference to the methods belonging to the `yarp::dev::IMap2D` interface is shown below (see http://www.yarp.it/classyarp_1_1dev_1_1IMap2D.html for a complete documentation).

`yarp::dev::Map2DClient` inherits from `yarp::dev::IMap2D`. It implements the methods required to communicate with a `yarp::dev::Map2DServer`.

```
class YARP_dev_API yarp::dev::IMap2D
{
    public:
    bool clear () = 0;
    bool store_map(const MapGrid2D& map) = 0;
    bool get_map(string map_name, MapGrid2D& map) =0;
    bool get_map_names(vector<string>& map_names) = 0;
    bool remove_map(string map_name) = 0;
    bool storeLocation(string location_name, Map2DLocation loc) = 0;
    bool getLocation(string location_name, Map2DLocation& loc) = 0;
    bool getLocationsList(vector<string>& locations) = 0;
    bool deleteLocation(string location_name) = 0;
    bool clearAllLocations() = 0;
};
```

VI RETRIEVE MAP EXAMPLE

The following code snippet, taken from the source code of robotPathPlanner module, shows how to retrieve a map from the `yarp::dev::Map2DServer` storage using the `yarp::dev::IMap2D` interface. The server needs to be externally launched before the execution of this example.

```

//...

IMap2D*                      m_iMap=0;
yarp::dev::MapGrid2D         m_current_map;
yarp::dev::Map2DLocation     m_localization_data;

//...

//open a map2DClient device
Property map_options;
map_options.put("device", "map2DClient");
map_options.put("local", "/robotPathPlanner");
map_options.put("remote", "/mapServer");
if (m_pMap.open(map_options) == false)
{
    yError() << "Unable␣to␣open␣mapClient";
    return false;
}

//get a IMap2D interface from the map2DClient device
m_pMap.view(m_iMap);
if (m_iMap == 0)
{
    yError() << "Unable␣to␣open␣map␣interface";
    return false;
}

//...
//get a map previously stored into the mapServer
bool map_get_succesfull = this->m_iMap->get_map(m_localization_data.map_id, m_current_map);
if (map_get_succesfull)
```

```
{
  //...
}
else
{
  yError() << "Unable␣to␣get␣map␣'" << m_localization_data.map_id << "'␣from␣map␣server";
  //...
}
```

VII INAVIGATION2D INTERFACE

A quick reference to the methods belonging to the `yarp::dev::INavigation2D` interface is shown below (see http://www.yarp.it/classyarp_1_1dev_1_1INavigation2D.html for a complete documentation).

A `yarp::dev::Navigation2DClient` implements methods from `INavigation2D` interface by forwarding the corresponding requests to different entities. Commands which operate on *Map2DLocations* (get/store/clear etc.) are forwarded to a connected *Map2DServer*. Navigation commands (stop, suspend, gotoTarget, etc.) are handled by a navigation module, such as robotPathPlanner. Depending on the chosen navigation module, some of these methods may return false (because they are not implemented) or they may forward the request to another module. For example, a local navigation module such as robotGoto does not implement gotoTargetByLocationName() since the method execution requires a global knowledge of the environment. Finally, methods related to localization (getCurrentPosition, setInitialPose) are handled by a localization module such as *LocalizationServer*.

```
class yarp::dev::INavigation2D
{
    public:
    bool gotoTargetByAbsoluteLocation(Map2DLocation loc) = 0;
    bool gotoTargetByLocationName(string location_name) = 0;
    bool gotoTargetByRelativeLocation(double x, double y, double theta) = 0;
    bool getCurrentPosition(Map2DLocation& loc) = 0;
    bool setInitialPose(Map2DLocation& loc) = 0;
    bool getAbsoluteLocationOfCurrentTarget(Map2DLocation& loc) = 0;
    bool getRelativeLocationOfCurrentTarget(double& x, double& y, double& theta) = 0;
    bool storeCurrentPosition(string location_name) = 0;
    bool storeLocation(string location_name, Map2DLocation loc) = 0;
    bool getLocation(string location_name, Map2DLocation& loc) = 0;
    bool getLocationsList(vector<string>& locations) = 0;
    bool deleteLocation(string location_name) = 0;
    bool clearAllLocations() = 0;
    bool getNavigationStatus(NavigationStatusEnum& status) = 0;
    bool stopNavigation() = 0;
    bool suspendNavigation() = 0;
    bool resumeNavigation() = 0;
};
```

VIII NAVIGATION STATUS ENUM

A complete list of `yarp::dev::INavigation2D::NavigationStatusEnum` is shown in the following table.

NavigationStatusEnum	Notes
navigation_status_idle	The robot is idle, waiting for a navigation command.
navigation_status_preparing_before_move	The robot is executing custom actions defined by the user before starting the navigation task.
navigation_status_moving	The robot is currently navigating towards the goal.
navigation_status_waiting_obstacle	The robot is currently avoiding an obstacle or it is waiting for external help. A watchdog is typically used to limit the maximum waiting time. If the timeout expires, the navigation status is changed to navigation_status_failing or navigation_status_aborted.
navigation_status_goal_reached	The robot has reached its current goal and it is about to change its navigation status to navigation_status_idle.
navigation_status_aborted	The path planner detected that there are no valid paths to reach the commanded goal. The user is requested to stop the current navigation task to clear this error.
navigation_status_failing	The robot has tried unsuccessfully to avoid an obstacle. A path replanning is required. If no replanning is performed, navigation status switches to navigation_status_aborted.
navigation_status_paused	Navigation task has been paused on user request.
navigation_status_thinking	The path planner is currently computing the robot path. The system automatically switches to navigation_status_moving when the operation is complete.
navigation_status_error	An unforeseen event occurred during navigation or not all required modules are currently available (e.g. missing localization or sensor data)

Fig.S1 shows an example of transition diagram between the states defined by `NavigationStatusEnum`. In particular, the presented diagram refers to the finite-state machine implemented in `robotPathPlanner`. It must be noticed that different navigation modules may implement just a subset of these states (e.g. `robotGoto` local navigation module does not implement a planner algorithm, thus the `thinking` state is omitted). User navigation modules can also extend this list of statuses by adding new definitions. However, even if a certain level of freedom is allowed for the internal statuses of newly developed navigation algorithms, it is recommended to adhere to the suggested conventions when communicating with external modules. This will maximize the integration with already existing YARP applications.

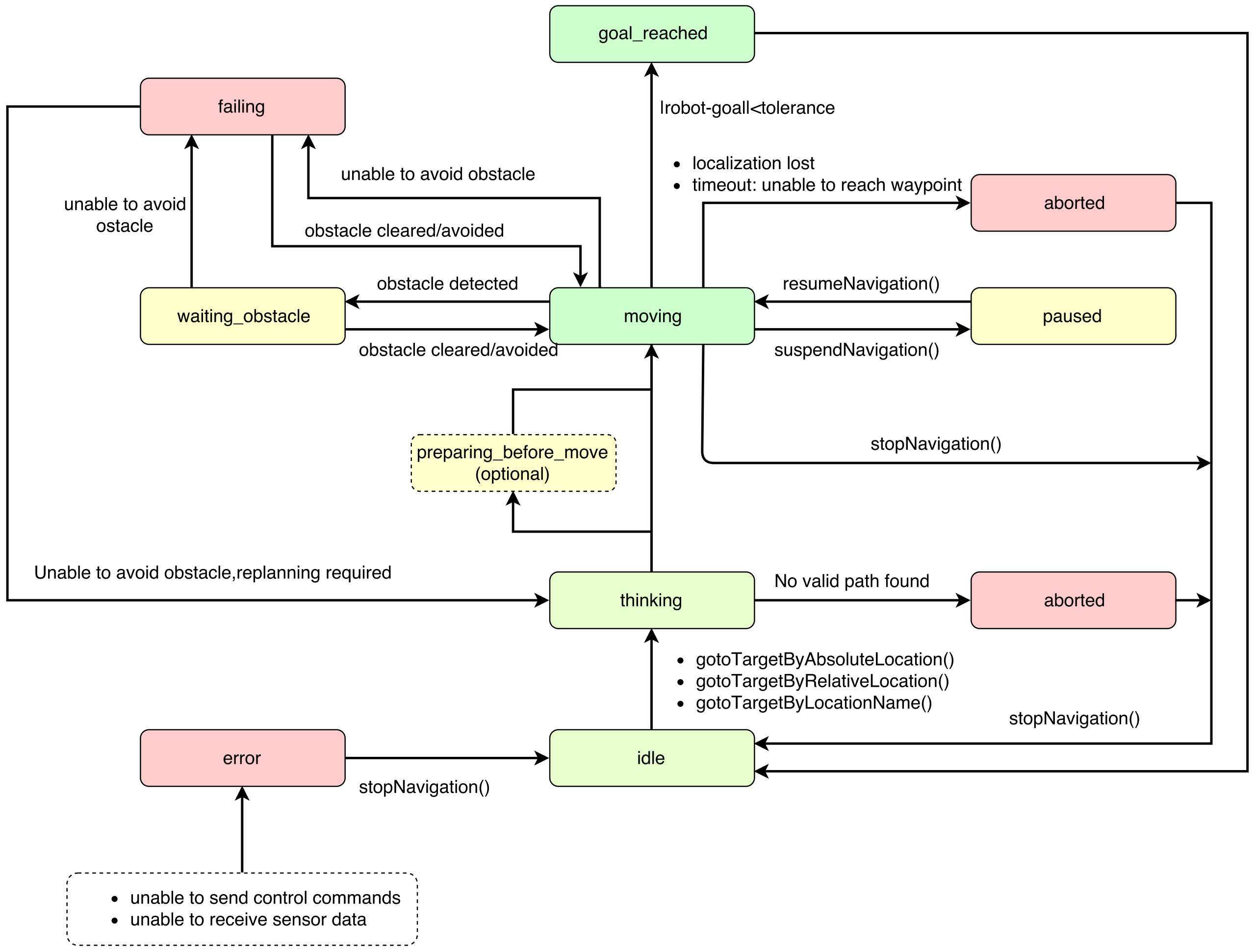

Figure S1: Finite-state machine for robotPathPlanner navigation module.

IX NAVIGATION2DCLIENT EXAMPLE

The following code snippet shows an example of usage of the two interfaces: `yarp::dev::IMap2D` and `yarp::dev::INavigation2D`. In order to execute successfully, this example assumes that:

- A mobile robot, either real or simulated, is available. *baseControl* module must be started and connected to the low-level motor/sensor interfaces provided by *YarpRobotInterface*. Examples included in robotology/navigation repository show how to simulate a simple wheeled robot able to move in a 2D world without using an external physics simulator such as Gazebo.
- A navigation module, such as `RobotPathPlanner`, is running. The module has to be connected to the robot control port (e.g. `/baseControl/control:i`). This port may belong either to a real or to a simulated robot.
- The two modules `LocalizationServer` and `Map2DServer` have been previously launched by the user. This allows the example to open the two PolyDrivers `yarp::dev::navigation2Client` and `yarp::dev::Map2DClient`.

In order to better clarify the execution flow of the example, a sequence diagram is provided in Section x, Fig.S2. Please note that for sake of clarity, the diagram omits all the operations performed by the path planner module `RobotPathPlanner`. This latter communicates with a local navigation module (e.g.`robotGoto`), which in turn is connected to the robot control module `baseControl`.

The diagram highlights the messages exchanged between the clients opened by the user application and the server modules (i.e. `localizationServer`, `Map2DServer` and `RobotPathPlanner`). In particular, it is should be noticed that there are cases in which a single command, such as `gotoTargetLocationbyName()`, is processed by a client by generating multiple requests. Depending on the command, these requests might be processed sequentially by the same server or by different ones: in the case of gotoTargetLocationbyName(), Map2DServer returns the location coordinates, while RobotPathPlanner computes the path.

A final consideration regards error handling. Even if this minimalistic example does not take in account failure cases, issues may occur both on client side (e.g. the server is not connected or no answer is received) and on server side (the user is commanding an invalid operation, such as trying to reaching a non-existing location). User is thus recommended to always check the value returned by the interface methods.

```
#include <yarp/os/all.h>
#include <yarp/dev/all.h>
#include <yarp/dev/INavigation2D.h>
#include <yarp/dev/IMap2D.h>

using namespace yarp::os;
using namespace yarp::dev;
using namespace std;

int main(int argc, char *argv[])
{
    Network yarp;

    //opens a navigation2DClient device and gets a INavigation2D interface.
    Property navTestCfg;
    navTestCfg.put("device",                "navigation2DClient");
    navTestCfg.put("local",                 "/navigationTest_navClient");
    navTestCfg.put("navigation_server",     "/robotPathPlanner");
    navTestCfg.put("map_locations_server", "/mapServer");
    navTestCfg.put("localization_server",  "/localizationServer");
    yarp::dev::PolyDriver     ddNavClient;
    yarp::dev::INavigation2D* iNav                 = 0;
    bool okClient              = ddNavClient.open(navTestCfg);
    bool okView                = ddNavClient.view(iNav);
    if(!okClient || !okView)
    {
        yError("Error␣opening␣INavigation2D␣interface");
        return false;
    }

    //opens a map2DClient device and gets a iMap2D interface.
    Property map_options;
    map_options.put("device", "map2DClient");
    map_options.put("local", "/navigationTest_mapClient");
```

```
    map_options.put("remote", "/mapServer");
    yarp::dev::PolyDriver ddMapClient;
    yarp::dev::IMap2D* iMap                  = 0;
    okClient             = ddMapClient.open(map_options);
    okView               = ddMapClient.view(iMap);
    if(!okClient || !okView)
    {
        yError("Error␣opening␣IMap2D␣interface");
        return false;
    }

    //Stops the navigation server, if it is executing some previosuly launched task.
    iNav->stopNavigation();

    //defines a new location called location_1 and saves it to map server
    Map2DLocation location_1;
    location_1.map_id="testMap";
    location_1.x=2.0;
    location_1.x=1.0;
    location_1.theta=45.0;
    iMap->storeLocation("location_1",location_1);

    //starts the navigation task
    iNav->gotoTargetByLocationName("location_1");

    double init_time = Time::now();
    NavigationStatusEnum status;
    const double TIMEOUT = 30.0; //seconds
    do
    {
        //gets the current position of the robot and displays it
        Map2DLocation current_position;
        iNav->getCurrentPosition(current_position);
        yInfo() << "Current␣robot␣position␣is:␣" << current_position.toString();

        //gets the navigation status
        iNav->getNavigationStatus(status);
        if(!iNav->getNavigationStatus(status))
        {
            yError() << "Unable␣to␣get␣navigation␣status";
            break;
        }

        //continue navigation until the goal is reached (or the timeout is expired)
        if (status == navigation_status_goal_reached)
        {
            yInfo() << "Goal␣reached!";
            break;
        }
        else if(Time::now() - init_time >= TIMEOUT)
        {
            yError() << "Timeout␣while␣heading␣towards␣current␣waypoint";
            break;
        }
```

```
        //sleep
        yarp::os::Time::delay(0.1);
    }
    while (1);

    //closes the opened device drivers
    ddNavClient.close();
    ddMapClient.close();

    return 0;
}
```

X ADDITIONAL FIGURES

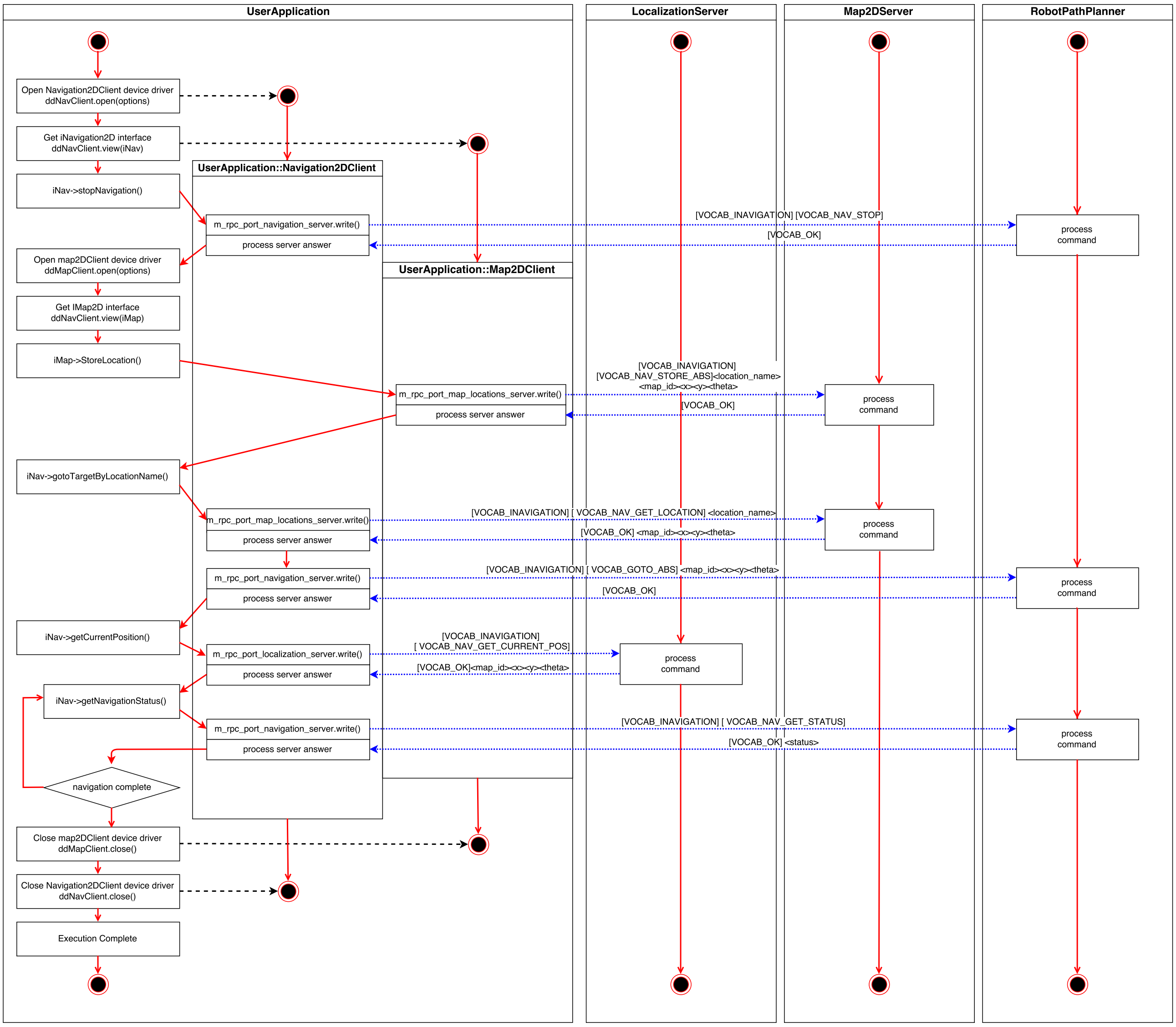

Figure S2: Sequence diagram for the Navigation2DClient example.

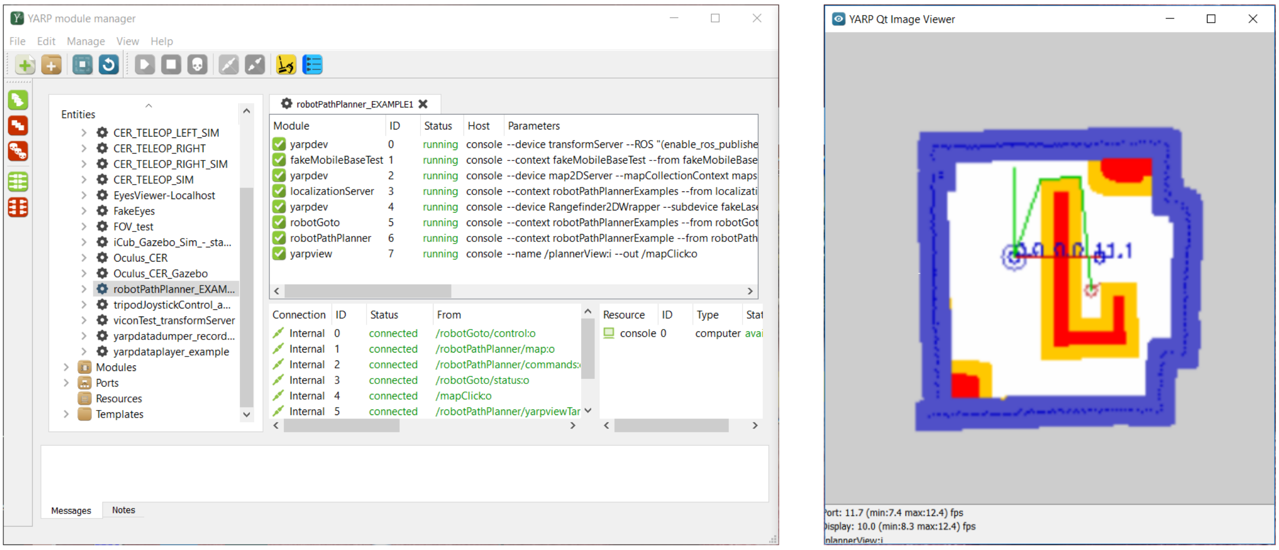

Figure S3: Left: YarpManager interface used to launch YARP applications. Right: Graphical output for RobotPathPlannerExample1 application. The image, generated by RobotPathPlanner, displays the map, the current robot position, the detected obstacles, the computed path to the goal.
